# A Cheap Disinfectant

**Published:** 1897-02

**Authors:** 


### A Cheap Disinfectant.

Nitrate of lead is the cheapest disinfectant known that fulfills its intent. It does not, however, prevent putrefaction. The chloride of lead is much more effective in all directions. It is made by dissolving a small teaspoonful of the nitrate of lead in a pint of boiling water, then dissolving two teaspoonfuls of common salt in eight quarts of water.

When both are thoroughly dissolved mix the solution. When the sediments have settled you have two gallons of clear fluid, which is a saturated solution of chloride of lead in water. A pound of nitrate of lead will make several barrels of the liquid and cost from eighteen to twenty-five cents retail.—*Annals of Hygiene.*

